# Comparison of Postoperative Pain and Function in Robotic Total Knee Arthroplasty and Conventional Total Knee Arthroplasty Amongst Patients at King Fahad Medical City in Riyadh, Saudi Arabia

**DOI:** 10.7759/cureus.36285

**Published:** 2023-03-17

**Authors:** Razan Alshatwi, Shoog Alfadhel, Mohammed Alrasheed, Abdulaziz Alhakbani, Osama AlShaya

**Affiliations:** 1 Orthopedic Surgery, King Fahad Medical City, Riyadh, SAU; 2 Orthopaedic Surgery, King Fahad Medical City, Riyadh, SAU; 3 Reconstructive Orthopaedic Department, King Fahad Medical City, Riyadh, SAU

**Keywords:** robotic surgical procedures, postoperative pain, robotic tka, conventional tka, knee osteo-arthritis, post-op pain, total knee replacement (tkr)

## Abstract

Background

Total knee arthroplasty (TKA) is the definitive surgical treatment for end-stage osteoarthritis and has been proven to relieve pain and improve function. With the rise in demand and the number of TKA procedures every year, more studies have been conducted on robotic TKA.

Objective

The objective of this study is to compare the postoperative pain between robotic and conventional TKA and the postoperative functional level between robotic and conventional TKA.

Method

This is a quantitative, observational, prospective study conducted from February 2022 to August 2022 amongst patients in the orthopaedic department of King Fahad Medical City, Riyadh, Saudi Arabia, who have undergone primary TKA for end-stage osteoarthritis using robotic TKA and conventional TKA. After applying the exclusion and inclusion criteria, a total of 26 patients (12 robotic and 14 conventional) were included in the study. The patients were assessed at three time points: two weeks, six weeks, and three months post-op. They were assessed using the Western Ontario and McMaster Universities Arthritis Index (WOMAC) score and the visual analogue scores (VAS) used to assess pain.

Result

A total of 26 patients were included in this research. The patients were divided into two groups: 12 robotic TKA patients and 14 conventional TKA patients. In this study, while comparing patients who underwent robotic TKA with those who underwent conventional TKA, no statistical significance was found regarding pain and function at all stages postoperatively.

Conclusion

There was no short-term difference between robotic and conventional TKA regarding pain and function. There is a need for further extensive research on robotic TKA in terms of cost-effectiveness, complications, implant survivorship, and long-term outcomes.

## Introduction

Total knee arthroplasty (TKA) is the definitive surgical treatment for end-stage osteoarthritis and has been proven to relieve pain and improve function [1,2]. TKA is performed in over 90,000 patients per year in the United Kingdom alone [1,3]. However, its satisfaction rate is lower compared to total hip arthroplasty (THA) [4]. Although with advanced implant design and material, studies show about 20% of TKA patients are dissatisfied [5].

The robotic TKA group underwent preoperative CT imaging to implement accurate size and placement. Intraoperative light-emitting diode trackers were used to trace the femur and tibia. The tracker guides the semi-active robotic system to achieve meticulous bone cuts. However, the patients who went for conventional TKA do not require CT. Moreover, conventional TKA used an intraoperative intramedullary guide in the femur and an extramedullary guide in the tibia for the bone cuts.

With the rise in demand and the number of TKAs performed each year, more studies have been conducted on robotic TKA. Research showed a decrease in postoperative pain, pain medication usage, and length of hospital stay in patients who underwent robotic TKA [6]. Acute postoperative pain is defined as the pain that is experienced within the first seven days after the procedure, and if the pain lasts for more than three months, it is considered chronic postoperative pain. Moreover, studies have analyzed alignment in robotic TKA and shown that it is superior to conventional TKA [7,8,9]. Component alignment in TKA is fundamental for a range of motion and weight load. Thus, malalignment can lead to implant failure [7,8].

Robotic TKA uses a preoperative 3D CT of the patient's native knee for a specific patient calculation. Robotic TKA can achieve precise bone cuts and an ideal implant size and positioning [9,10]. Furthermore, it gives balanced gaps in flexion and extension [11]. Robotic TKA also has a role in minimizing soft tissue damage, which has a clear correlation with pain [12]. In this study, we aimed to compare early postoperative pain and functional level in robotic TKA with those in conventional TKA.

## Materials and methods

Research methodology

This quantitative, observational, prospective study was conducted from February 2022 to August 2022 amongst patients in the orthopaedic department of King Fahad Medical City, Riyadh, Saudi Arabia, who had undergone primary TKA for end-stage osteoarthritis using robotic TKA and conventional TKA. All the procedures were performed by the same surgeon at the same institution. Also, the procedures had the same approach throughout this study. This research involved 10 to 15 patients from each group from the same period and compared pain and function post-op. Twelve subjects were operated on via robotic TKA, while conventional TKA was used in 14. For both groups, posterior-stabilized (PS) prostheses from the same company were used.

We have all patients with end-stage osteoarthritis (representing grade four in the Kellgren-Lawrence classification system) who have undergone unilateral TKA. MAKOplasty® (Mako) robotic TKA was used in 12 patients, while 14 patients underwent conventional TKA. We excluded all the patients that underwent revision TKA, bilateral TKA, unicompartmental arthroplasty, and patients with postoperative infection. The patients’ ages ranged from 50 to 75 years old without regard to gender.

The robotic TKA group underwent preoperative CT imaging to implement accurate size and placement. Intraoperative light-emitting diode trackers were used to trace the femur and tibia. Since there is a lot of radiation exposure in this procedure, informed consent was obtained from all patients after an explanation of the risks and benefits of the procedure. The tracker guides the semi-active robotic system to achieve meticulous bone cuts. However, the patients who went for conventional TKA do not require CT. Moreover, conventional TKA used an intraoperative intramedullary guide in the femur and an extramedullary guide in the tibia for the bone cuts.

The patients were assessed at three time points: two weeks, six weeks, and 12 weeks post-op. They were assessed using the Western Ontario and McMaster Universities Arthritis Index (WOMAC) score (Appendix 1) and visual analogue scores (VAS) used to assess pain. The data were collected in the clinic during the patients’ regular follow-ups using WOMAC and VAS as measurement tools.

Statistical analysis

Microsoft Excel was used to collect all the variables. The t-test was utilized to compare the two groups. The results were considered statistically significant if the p-value < 0.05.

Ethical considerations

There was a consent form used for all the participants. It recorded the participants' information, and all the information was treated with confidentiality. Moreover, there was no enclosure of personal information throughout the research. The patients were informed about the benefits, complications, and expected management course. The consent forms were provided in Arabic and English. Informed consent was obtained from each participant prior to data collection after explaining the study objectives. Data collection started after getting ethical approval (number FWA00018774) from King Fahad Medical City Research Center.

## Results

A total of 26 patients were included in our research. Two patients were excluded as they were lost to follow-up. The groups included patients who underwent robotic TKA (12) and those who underwent conventional TKA (14); the mean ages were from 66 to 68 years (Table 1).

**Table 1:** Age and gender distribution of patients TKA: total knee arthroplasty

		Conventional TKA N(%)	Robotic TKA N(%)	p-value
Age (year)	Min-max	54 - 80	55 - 85	0.403
	Mean ± SD	66.1 ± 7.8	68.3 ± 8.2	
	Median (P_25_, P_75_)	66 (60, 71)	68 (62, 73)	
Gender	Male	3 (21.4)	4 (33.3)	0.665
	Female	11 (78.6)	8 (66.7)	

When comparing patients who underwent robotic TKA with patients who underwent conventional TKA, no statistical significance was found regarding pain and function at all stages postoperatively (Table 2). Concerning the patients’ ability to get in and out of the bath, the conventional group had a better outcome than the robotic group at week 12 (p-value=0.033). The two groups experienced an improvement in pain and function with time when compared at weeks two, six, and 12 postoperatively.

**Table 2:** Robotic versus conventional total knee arthroplasty (TKA): postoperative pain and function at two weeks, six weeks, and 12 weeks *VAS: visual analogue scale; WQ: represents each question in the attached survey (Western Ontario and McMaster Universities Arthritis Index (WOMAC) score)

x		Week Two		p-value	Week Six		p-value	Week 12		p-value
		Conventional TKA	Robotic TKA		Conventional TKA	Robotic TKA		Conventional TKA	Robotic TKA	
WQ1	None	1 (7.1)	0 (.0)	0.89	3 (21.4)	0 (.0)	0.276	8 (57.1)	5 (41.7)	0.394
	Slight	1 (7.1)	3 (25.0)		3 (21.4)	6 (50.0)		5 (35.7)	5 (41.7)	
	Moderate	3 (21.4)	2 (16.7)		6 (42.9)	4 (33.3)		1 (7.1)	0 (.0)	
	Severe	4 (28.6)	3 (25.0)		1 (7.1)	2 (16.7)		0 (.0)	2 (16.7)	
	Extreme	5 (35.7)	4 (33.3)		1 (7.1)	0 (.0)		0 (.0)	0 (.0)	
WQ2	None	0 (.0)	1 (8.3)	0.442	2 (14.3)	0 (.0)	0.103	5 (35.7)	6 (50.0)	0.667
	Slight	0 (.0)	2 (16.7)		1 (7.1)	6 (50.0)		6 (42.9)	2 (16.7)	
	Moderate	5 (35.7)	2 (16.7)		8 (57.1)	4 (33.3)		2 (14.3)	2 (16.7)	
	Severe	3 (21.4)	2 (16.7)		2 (14.3)	1 (8.3)		1 (7.1)	1 (8.3)	
	Extreme	6 (42.9)	5 (41.7)		1 (7.1)	1 (8.3)		0 (.0)	1 (8.3)	
WQ3	None	1 (7.1)	1 (8.3)	0.251	5 (35.7)	5 (41.7)	0.83	11 (78.6)	10 (83.3)	1
	Slight	0 (.0)	1 (8.3)		3 (21.4)	4 (33.3)		1 (7.1)	1 (8.3)	
	Moderate	4 (28.6)	6 (50.0)		5 (35.7)	3 (25.0)		2 (14.3)	1 (8.3)	
	Severe	3 (21.4)	3 (25.0)		0 (.0)	0 (.0)		0 (.0)	0 (.0)	
	Extreme	6 (42.9)	1 (8.3)		1 (7.1)	0 (.0)		0 (.0)	0 (.0)	
WQ4	None	3 (21.4)	3 (25.0)	0.801	7 (50.0)	5 (41.7)	0.533	12 (85.7)	10 (83.3)	0.781
	Slight	2 (14.3)	1 (8.3)		3 (21.4)	2 (16.7)		2 (14.3)	1 (8.3)	
	Moderate	4 (28.6)	3 (25.0)		2 (14.3)	5 (41.7)		0 (.0)	1 (8.3)	
	Severe	2 (14.3)	4 (33.3)		1 (7.1)	0 (.0)		0 (.0)	0 (.0)	
	Extreme	3 (21.4)	1 (8.3)		1 (7.1)	0 (.0)		0 (.0)	0 (.0)	
WQ5	None	5 (35.7)	2 (16.7)	0.557	5 (35.7)	3 (25.0)	0.432	11 (78.6)	8 (66.7)	0.809
	Slight	1 (7.1)	3 (25.0)		3 (21.4)	6 (50.0)		3 (21.4)	3 (25.0)	
	Moderate	3 (21.4)	3 (25.0)		3 (21.4)	3 (25.0)		0 (.0)	1 (8.3)	
	Severe	2 (14.3)	3 (25.0)		3 (21.4)	0 (.0)		0 (.0)	0 (.0)	
	Extreme	3 (21.4)	1 (8.3)		0 (.0)	0 (.0)		0 (.0)	0 (.0)	
WQ6	None	2 (14.3)	4 (33.3)	0.467	3 (21.4)	5 (41.7)	0.655	9 (64.3)	7 (58.3)	0.92
	Slight	2 (14.3)	0 (.0)		5 (35.7)	4 (33.3)		3 (21.4)	3 (25.0)	
	Moderate	4 (28.6)	4 (33.3)		5 (35.7)	2 (16.7)		1 (7.1)	2 (16.7)	
	Severe	1 (7.1)	2 (16.7)		1 (7.1)	1 (8.3)		1 (7.1)	0 (.0)	
	Extreme	5 (35.7)	2 (16.7)		0 (.0)	0 (.0)		0 (.0)	0 (.0)	
WQ7	None	2 (14.3)	5 (41.7)	0.378	3 (21.4)	6 (50.0)	0.279	8 (57.1)	7 (58.3)	1
	Slight	2 (14.3)	0 (.0)		5 (35.7)	4 (33.3)		4 (28.6)	3 (25.0)	
	Moderate	4 (28.6)	3 (25.0)		5 (35.7)	1 (8.3)		1 (7.1)	2 (16.7)	
	Severe	1 (7.1)	2 (16.7)		1 (7.1)	1 (8.3)		1 (7.1)	0 (.0)	
	Extreme	5 (35.7)	2 (16.7)		0 (.0)	0 (.0)		0 (.0)	0 (.0)	
WQ8	None	0 (.0)	1 (8.3)	0.161	2 (14.3)	3 (25.0)	0.374	5 (35.7)	7 (58.3)	0.14
	Slight	0 (.0)	2 (16.7)		1 (7.1)	3 (25.0)		7 (50.0)	1 (8.3)	
	Moderate	6 (42.9)	2 (16.7)		8 (57.1)	3 (25.0)		1 (7.1)	2 (16.7)	
	Severe	2 (14.3)	4 (33.3)		2 (14.3)	3 (25.0)		1 (7.1)	2 (16.7)	
	Extreme	6 (42.9)	3 (25.0)		1 (7.1)	0 (.0)		0 (.0)	0 (.0)	
WQ9	None	0 (.0)	1 (8.3)	0.161	2 (14.3)	3 (25.0)	0.374	6 (42.9)	7 (58.3)	0.43
	Slight	0 (.0)	2 (16.7)		1 (7.1)	3 (25.0)		5 (35.7)	1 (8.3)	
	Moderate	6 (42.9)	2 (16.7)		8 (57.1)	3 (25.0)		2 (14.3)	2 (16.7)	
	Severe	2 (14.3)	4 (33.3)		2 (14.3)	3 (25.0)		1 (7.1)	2 (16.7)	
	Extreme	6 (42.9)	3 (25.0)		1 (7.1)	0 (.0)		0 (.0)	0 (.0)	
WQ10	None	2 (14.3)	4 (33.3)	0.419	7 (50.0)	7 (58.3)	0.75	9 (64.3)	7 (58.3)	0.867
	Slight	3 (21.4)	1 (8.3)		3 (21.4)	1 (8.3)		3 (21.4)	2 (16.7)	
	Moderate	4 (28.6)	1 (8.3)		4 (28.6)	4 (33.3)		2 (14.3)	3 (25.0)	
	Severe	5 (35.7)	5 (41.7)		0 (.0)	0 (.0)		0 (.0)	0 (.0)	
	Extreme	0 (.0)	1 (8.3)		0 (.0)	0 (.0)		0 (.0)	0 (.0)	
WQ11	None	4 (28.6)	3 (25.0)	0.27	8 (57.1)	7 (58.3)	1	10 (71.4)	10 (83.3)	0.791
	Slight	2 (14.3)	0 (.0)		2 (14.3)	1 (8.3)		3 (21.4)	1 (8.3)	
	Moderate	4 (28.6)	3 (25.0)		3 (21.4)	4 (33.3)		1 (7.1)	1 (8.3)	
	Severe	1 (7.1)	5 (41.7)		1 (7.1)	0 (.0)		0 (.0)	0 (.0)	
	Extreme	3 (21.4)	1 (8.3)		0 (.0)	0 (.0)		0 (.0)	0 (.0)	
WQ12	None	5 (35.7)	4 (33.3)	1	8 (57.1)	7 (58.3)	0.543	11 (78.6)	10 (83.3)	1
	Slight	2 (14.3)	1 (8.3)		3 (21.4)	1 (8.3)		1 (7.1)	1 (8.3)	
	Moderate	2 (14.3)	2 (16.7)		2 (14.3)	4 (33.3)		2 (14.3)	1 (8.3)	
	Severe	4 (28.6)	5 (41.7)		1 (7.1)	0 (.0)		0 (.0)	0 (.0)	
	Extreme	1 (7.1)	0 (.0)		0 (.0)	0 (.0)		0 (.0)	0 (.0)	
WQ13	None	12 (85.7)	10 (83.3)	0.781	10 (71.4)	10 (83.3)	0.791	12 (85.7)	11 (91.7)	1
	Slight	2 (14.3)	1 (8.3)		3 (21.4)	1 (8.3)		2 (14.3)	1 (8.3)	
	Moderate	0 (.0)	0 (.0)		1 (7.1)	1 (8.3)		0 (.0)	0 (.0)	
	Severe	0 (.0)	1 (8.3)		0 (.0)	0 (.0)		0 (.0)	0 (.0)	
	Extreme	0 (.0)	0 (.0)		0 (.0)	0 (.0)		0 (.0)	0 (.0)	
WQ14	None	4 (28.6)	2 (16.7)	3194	9 (64.3)	7 (58.3)	0.867	11 (78.6)	10 (83.3)	1
	Slight	2 (14.3)	3 (25.0)		2 (14.3)	3 (25.0)		2 (14.3)	1 (8.3)	
	Moderate	3 (21.4)	1 (8.3)		3 (21.4)	2 (16.7)		1 (7.1)	1 (8.3)	
	Severe	2 (14.3)	6 (50.0)		0 (.0)	0 (.0)		0 (.0)	0 (.0)	
	Extreme	3 (21.4)	0 (.0)		0 (.0)	0 (.0)		0 (.0)	0 (.0)	
WQ15	None	10 (71.4)	10 (83.3)	0.125	12 (85.7)	10 (83.3)	0.781	13 (92.9)	10 (83.3)	0.203
	Slight	0 (.0)	2 (16.7)		1 (7.1)	2 (16.7)		0 (.0)	2 (16.7)	
	Moderate	0 (.0)	0 (.0)		1 (7.1)	0 (.0)		1 (7.1)	0 (.0)	
	Severe	3 (21.4)	0 (.0)		0 (.0)	0 (.0)		0 (.0)	0 (.0)	
	Extreme	1 (7.1)	0 (.0)		0 (.0)	0 (.0)		0 (.0)	0 (.0)	
WQ16	None	4 (28.6)	4 (33.3)	0.676	8 (57.1)	7 (58.3)	1	13 (92.9)	10 (83.3)	0.72
	Slight	2 (14.3)	2 (16.7)		3 (21.4)	3 (25.0)		0 (.0)	1 (8.3)	
	Moderate	4 (28.6)	1 (8.3)		3 (21.4)	2 (16.7)		1 (7.1)	1 (8.3)	
	Severe	4 (28.6)	4 (33.3)		0 (.0)	0 (.0)		0 (.0)	0 (.0)	
	Extreme	0 (.0)	1 (8.3)		0 (.0)	0 (.0)		0 (.0)	0 (.0)	
WQ17	None	6 (42.9)	4 (33.3)	0.89	9 (64.3)	8 (66.7)	1	11 (78.6)	9 (75.0)	1
	Slight	2 (14.3)	1 (8.3)		3 (21.4)	2 (16.7)		1 (7.1)	1 (8.3)	
	Moderate	3 (21.4)	2 (16.7)		2 (14.3)	2 (16.7)		2 (14.3)	2 (16.7)	
	Severe	3 (21.4)	4 (33.3)		0 (.0)	0 (.0)		0 (.0)	0 (.0)	
	Extreme	0 (.0)	1 (8.3)		0 (.0)	0 (.0)		0 (.0)	0 (.0)	
WQ18	None	3 (21.4)	4 (33.3)	0.436	8 (57.1)	8 (66.7)	1	13 (92.9)	9 (75.0)	0.44
	Slight	1 (7.1)	2 (16.7)		3 (21.4)	2 (16.7)		0 (.0)	2 (16.7)	
	Moderate	5 (35.7)	1 (8.3)		3 (21.4)	2 (16.7)		1 (7.1)	1 (8.3)	
	Severe	5 (35.7)	4 (33.3)		0 (.0)	0 (.0)		0 (.0)	0 (.0)	
	Extreme	0 (.0)	1 (8.3)		0 (.0)	0 (.0)		0 (.0)	0 (.0)	
WQ19	None	7 (50.0)	4 (33.3)	0.735	9 (64.3)	8 (66.7)	0.728	13 (92.9)	9 (75.0)	0.379
	Slight	2 (14.3)	1 (8.3)		4 (28.6)	2 (16.7)		0 (.0)	1 (8.3)	
	Moderate	2 (14.3)	3 (25.0)		1 (7.1)	2 (16.7)		1 (7.1)	2 (16.7)	
	Severe	3 (21.4)	3 (25.0)		0 (.0)	0 (.0)		0 (.0)	0 (.0)	
	Extreme	0 (.0)	1 (8.3)		0 (.0)	0 (.0)		0 (.0)	0 (.0)	
WQ20	None	3 (21.4)	5 (41.7)	0.272	9 (64.3)	7 (58.3)	0.212	13 (92.9)	8 (66.7)	0.033
	Slight	1 (7.1)	2 (16.7)		1 (7.1)	4 (33.3)		0 (.0)	4 (33.3)	
	Moderate	6 (42.9)	1 (8.3)		4 (28.6)	1 (8.3)		1 (7.1)	0 (.0)	
	Severe	4 (28.6)	4 (33.3)		0 (.0)	0 (.0)		0 (.0)	0 (.0)	
	Extreme	0 (.0)	0 (.0)		0 (.0)	0 (.0)		0 (.0)	0 (.0)	
WQ21	None	7 (50.0)	5 (41.7)	0.328	11 (78.6)	9 (75.0)	0.809	12 (85.7)	8 (66.7)	0.419
	Slight	3 (21.4)	0 (.0)		0 (.0)	1 (8.3)		2 (14.3)	2 (16.7)	
	Moderate	2 (14.3)	4 (33.3)		3 (21.4)	2 (16.7)		0 (.0)	2 (16.7)	
	Severe	2 (14.3)	3 (25.0)		0 (.0)	0 (.0)		0 (.0)	0 (.0)	
	Extreme	0 (.0)	0 (.0)		0 (.0)	0 (.0)		0 (.0)	0 (.0)	
WQ22	None	1 (7.1)	4 (33.3)	0.296	9 (64.3)	8 (66.7)	0.319	13 (92.9)	10 (83.3)	0.203
	Slight	2 (14.3)	2 (16.7)		1 (7.1)	3 (25.0)		0 (.0)	2 (16.7)	
	Moderate	6 (42.9)	2 (16.7)		4 (28.6)	1 (8.3)		1 (7.1)	0 (.0)	
	Severe	4 (28.6)	4 (33.3)		0 (.0)	0 (.0)		0 (.0)	0 (.0)	
	Extreme	1 (7.1)	0 (.0)		0 (.0)	0 (.0)		0 (.0)	0 (.0)	
WQ23	None	9 (64.3)	9 (75.0)	1	12 (85.7)	11 (91.7)	1	13 (92.9)	9 (75.0)	0.085
	Slight	1 (7.1)	0 (.0)		1 (7.1)	1 (8.3)		0 (.0)	3 (25.0)	
	Moderate	1 (7.1)	1 (8.3)		1 (7.1)	0 (.0)		1 (7.1)	0 (.0)	
	Severe	2 (14.3)	2 (16.7)		0 (.0)	0 (.0)		0 (.0)	0 (.0)	
	Extreme	1 (7.1)	0 (.0)		0 (.0)	0 (.0)		0 (.0)	0 (.0)	
WQ24	None	10 (71.4)	10 (83.3)	1	12 (85.7)	11 (91.7)	1	13 (92.9)	9 (75.0)	0.085
	Slight	1 (7.1)	0 (.0)		1 (7.1)	1 (8.3)		0 (.0)	3 (25.0)	
	Moderate	0 (.0)	1 (8.3)		1 (7.1)	0 (.0)		1 (7.1)	0 (.0)	
	Severe	2 (14.3)	1 (8.3)		0 (.0)	0 (.0)		0 (.0)	0 (.0)	
	Extreme	1 (7.1)	0 (.0)		0 (.0)	0 (.0)		0 (.0)	0 (.0)	
VAS*	Min - max	5 - 9	4 - 9	1	1 - 7	0 - 9	0.462	0 - 4	0 - 6	0.432
	Mean ± SD	7.1 ± 1.4	7.1 ± 1.6		3.2 ± 1.7	4.1 ± 2.6		0.9 ± 1.4	1.6 ± 2	
	Median (P_25_, P_75_)	8 (6, 8)	7 (6, 9)		3 (2, 3)	4 (2, 6)		0 (0, 1)	1 (0, 3)	

## Discussion

In this study, the patients who underwent robotic TKA and conventional TKA showed similar results with regard to pain and function. The parameters from the VAS and WOMAC scores in both groups were equivalent after two weeks, six weeks, and 12 weeks. Although our result did not demonstrate any difference between the two groups, some studies have shown that patients who underwent robotic TKA had a lower rate of pain, analgesic use, and length of hospital stay [6,13]. In addition, some studies showed only short-term improvement < six months [14,15], whereas other studies showed significant outcomes in long-term follow-up [16].

Moreover, robotic TKA has become more prominent worldwide. However, even with the advanced precision that robotic TKA offers, some studies did not show it had superiority compared to conventional TKA [17,18,19]. One study reported that robotic TKA was longer and more expensive, with no difference in the rate of complications or length of hospital stay [19]. Another study that compared inflammatory markers between robotic and conventional TKA showed lower interleukin-6 (IL-6) in robotic TKA that can be explained by minimized soft tissue insult, yet it showed no difference in functional levels between the groups [20]. Furthermore, a study that observed the two groups in the long term (at least 10 years) showed no difference in functional outcomes or overall survivorship, and the robotic TKA group had a longer operative time [21]. Another comparative study done between robotic and conventional TKA showed that after a follow-up of 10 years, there was no difference in the long-term implant survival rate. However, the robotic technique had superior clinical and radiological outcomes than the conventional technique [22].

With regards to the cost-effectiveness of the robotic TKA, the Yechu Hua study showed that hospitals with a high number of patients delivered higher value in performing the robotic TKA by lowering both revision rates and post-op care costs [23]. The operation duration is one of the important factors when we measure postoperative pain since prolonged procedures can affect postoperative pain in an indirect way. In the literature, there is a lack of articles that compare the operation time between both robotic TKA and conventional TKA.

Limitations

Our study has several limitations, including a short follow-up as our study assessed up to 12 weeks postop. In addition, there was a limited sample size of patients that underwent robotic TKA during the data collection period. Also, our study did not include radiological assessments. However, there was no increased risk of complications observed among patients who underwent robotic TKA compared to those who underwent conventional TKA.

## Conclusions

In this study, we compared early postoperative pain and functional status in robotic TKA with those in conventional TKA. Our study, like many others, showed no short-term difference between robotic and conventional TKA regarding pain and function. However, there is a need for further extensive research on robotic TKA in terms of cost-effectiveness, complications, implant survival, and long-term outcomes. The limited number of patients who underwent robotic TKA was one of the main limitations we faced during this study. We recommend increasing the sample size in future studies to have more reliable results.

## Figures and Tables

**Figure 1:**
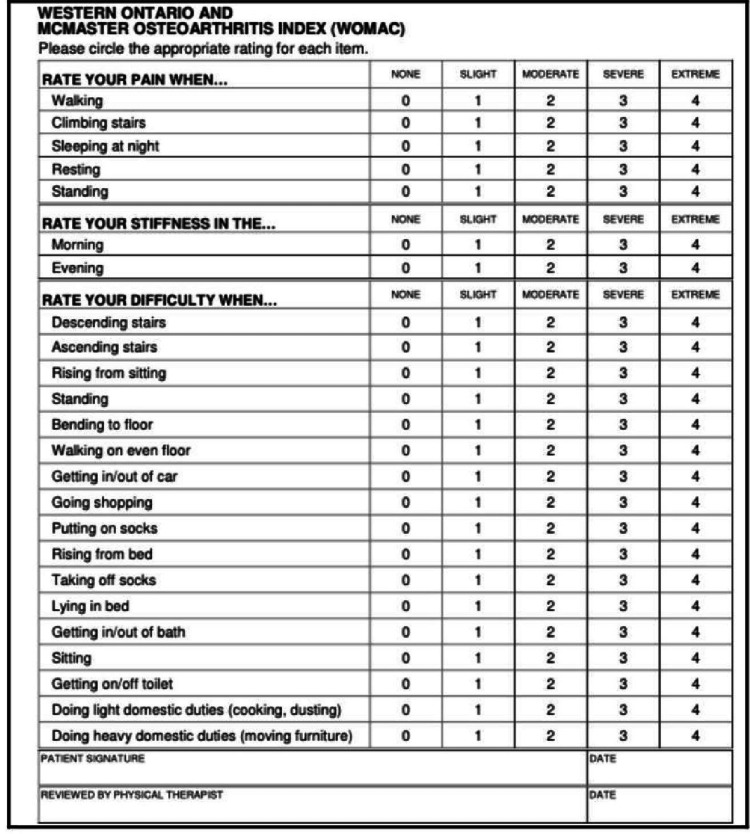
Western Ontario and McMaster Universities Arthritis Index (WOMAC) score
